# Early-Life Intestine Microbiota and Lung Health in Children

**DOI:** 10.1155/2017/8450496

**Published:** 2017-11-21

**Authors:** Giusy Ranucci, Vittoria Buccigrossi, Maiara Brusco de Freitas, Alfredo Guarino, Antonietta Giannattasio

**Affiliations:** ^1^Department of Translational Medical Science, Section of Pediatrics, University of Naples Federico II, Naples, Italy; ^2^Federal University of Santa Catarina, Florianopolis, SC, Brazil; ^3^Department of Medicine and Health Sciences, University of Molise, Campobasso, Italy

## Abstract

The gastrointestinal microbiota plays a critical role in nutritional, metabolic, and immune functions in infants and young children and has implications for future lung health status. Understanding the role of intestinal dysbiosis in chronic lung disease progression will provide opportunities to design early interventions to improve the course of the disease. Gut microbiota is established within the first 1 to 3 years of life and remains relatively stable throughout the life span. In this review, we report the recent development in research in gut-lung axis, with focus on the effects of targeting microbiota of infants and children at risk of or with progressive lung diseases. The basic concept is to exploit this approach in critical window to achieve the best results in the control of future health.

## 1. Introduction

Clinical research in the last decades focused on host-microbe crosstalk especially at intestinal level raising the hypothesis that the gut microbiota is one of the key factors that determine host health. The immune and metabolic functions are influenced by the colonization of intestine by friendly bacteria [1] and a growing number of diseases derive from the gut microbiota composition. The change in microbiota structure often starts in the early life in children with or at risk of chronic diseases, such as cystic fibrosis [2, 3].

The microbiome is established within the first 1 to 3 years of life and remains relatively stable throughout the life span [4]. The neonatal period is a critical window for immune programming that affects the global health status for a lifetime, and the development of the gut microbiota may help predicting specific disease risk and disease progression or elimination of disease altogether [5].

In this review, we analyze the main factors and phases of early microbial imprinting, gut-lung axis in the progression of chronic lung diseases in children, and the manipulation of early-life gut microbiota for modifying history of lung diseases in children.

## 2. Factors Influencing Gut Microbiota Composition in Early Infancy

Environmental factors together with genetics and immune system activity influence microbial colonization pattern in newborns Table 1. The concept that the fetus is sterile has been reconsidered after detection of microbial traces in fetal and maternal compartments [6, 7]. It is now believed that there is a prenatal colonization with vertically transferred microbial material.

The type of delivery influences the pattern of colonization in infant gut. Vaginally delivered infants are colonized with bacterial communities from the mother's vaginal and intestinal tract. Infants born to cesarean section lack such exposure, leading to the first establishment of bacteria similar to the human skin microbiota [8]. In addition, the intestinal colonization by *Lactobacillus*, *Bifidobacteria*, and *Bacteroides* in infants born by cesarean section is delayed [9].

Infant feeding influences the gut microbial composition with long-lasting effects. Breastfeeding has been associated with a variety of long-term beneficial effects including lower incidence of obesity [10], diabetes [11], and allergies [12, 13]. Breast milk is the gold standard in infant nutrition, because it is species specifically adapted to the infant nutritional needs and functional to drive the development of the immune system. In addition to nutritional support, breast milk provides bioactive constituents that promote the growth of a wide and dynamic array of microorganisms [14]. Colostrum has higher microbial diversity than transitional and mature milk, and factors influencing the microbial community in breast milk also depend on the mother's nutritional status and the type of delivery.

An infant gut microbiota dominated by *Bifidobacteria* has consistently been associated with host health. The predominance of *Bifidobacteria* in breastfed infant stools was firstly found over 100 years ago, suggesting that breast milk contains specific molecules that stimulate the growth of these bacteria, defined as bifidus factors [15]. Today, compelling evidence show that prebiotics in breastmilk promote the growth of *Bifidobacteria*. The latter dominate the microbiota of breastfed infants, whereas formula-fed infants had higher proportions of *Bacteroides* and members of the *Clostridium coccoides* and *Lactobacillus* groups [16]. In addition, breastfeeding modulates innate immune response [17].

Antibiotics have a major impact on gut microbiome composition particularly in young infants. In the latter, the gut bacteria structure is very unstable, and the continuous and repeated use of antibiotics, especially if the antibiotic used is a broad-spectrum one, profoundly alters gut microbial structure with major late clinical consequences. Recent papers show that prenatal use of antibiotics is associated with an increased risk of subsequent asthma [18].

Of course, antibiotics are often necessary for certain conditions, but their use should be considered in the light of the new data summarized in Table 2. Russell and colleagues found that perinatal antibiotic use exerts highly selective modifications on resident gut flora, which in turn lead to very specific alterations in susceptibility to TH2- or TH1-/TH17-driven lung inflammatory disease [19]. A recent study found that children lacking four bacteria types were more likely to develop asthma, but the only significant environmental factor among these children was receiving more antibiotics in their first year of life than children with lower asthma risk [18]. Early infantile infections including common intestinal infections do change intestinal microbial structure with long-lasting effects [20].

## 3. Gut-Lung Axis in the Progression of Chronic Lung Diseases in Children

The gut and respiratory epithelia provide a physical barrier against microbial penetration, and colonization with the normal microbiota generates resistance to pathogens. The role of microbiota in lung homeostasis and immunity is supported by the poor outcomes of germ-free mice that were exposed to acute infections [21] and their susceptibility to allergic airway disease [22]. Dysbiosis in the gut has recently been linked to alterations in immune responses and to disease development in the lungs. In the last few years, chronic lung diseases, such as asthma and cystic fibrosis, have been investigated to evaluate the potential role of intestinal dysbiosis in their development. Primarily, exacerbations of chronic gut and lung diseases share key conceptual features with the dysregulation of intestinal microbial ecosystem.

Cystic fibrosis (CF) is a disease in which recurrent and chronic infection leads to a progressive decline of lung function and ultimately to death. In observational studies in humans, CF has been associated with aberrant microbial colonization of the intestinal and the respiratory tracts. These findings are the likely consequences of the loss of CF transmembrane conductance regulator (CFTR) function and the resulting altered microenvironment [23]. Mutations in CFTR fundamentally affect the airway and intestinal microenvironment and result in abnormal colonization pattern of microorganisms in patients with CF even in the absence of antibiotic exposure [24]. More severe CFTR allelic variants such as homozygous DF508 correlate with more significant alterations in gut microbiome pattern. Chronic gut inflammation is seen in CF even in the absence of overt gastrointestinal symptoms and is thought to be a driver of systemic inflammation, a hallmark of the disease [25]. The association between gut microbial colonization in early life and respiratory outcomes in patients with CF has been investigated, and breastfeeding was associated with delayed exacerbations, gut diversity, and prolonged periods of well-being and specific bacterial communities in the gut prior to respiratory complications [26]. Bacteria in the respiratory tract in CF originate from intestinal microbiota and are thought to contribute to the dynamic interactions between the host and microbial communities in CF [27].

Studies have demonstrated also a relationship between poor nutrition and the development of bronchopulmonary dysplasia in preterm infants who require prolonged supplemental oxygen therapy [28]. No data are available regarding gut dysbiosis in this setting.

Children developing asthma have a reduced gut microbial diversity in the first year of life as compared to healthy children [29]. In particular, delivery mode has been associated with wheezing and asthma until school age, mediated by specific gut bacterial groups [30]. The presence of beneficial bacteria, such as *Bifidobacterium longum*, and a reduction in *Bacteroides fragilis* [31] in the gut have been associated with a lower incidence of asthma. The relative abundance of the bacterial genera *Lachnospira*, *Veillonella*, *Faecalibacterium*, and *Rothia* seems significantly decreased in children at risk of asthma, with consequent reduced levels of fecal acetate and dysregulation of enterohepatic metabolites [31].

## 4. Manipulation of Early-Life Gut Microbiota in Lung Diseases in Children

Oral administration of probiotics and prebiotics or a combination of both (the so-called “synbiotic approach”) could indirectly influence the composition of airway microbiota through the release of bacterial products or metabolites that reach the lung and promote the outgrowth of beneficial bacteria or directly, via microaspiration of the probiotic strain from the intestinal tract to the airways [32]. These mechanisms may restore a health-promoting microbiota and have a beneficial effect on the course of the disease. Moreover, few probiotic strains have a role in modifying the course of lung diseases. In animal studies, *Lactobacilli* have profound immunoregulatory effects on the lung, but the results of clinical trials in humans have been highly variable. Strain differences may in part explain the observed variability.

In humans, administration of *Lactobacillus* GG (LGG) negatively influenced the incidence of ventilator-associated pneumonia [33] and reduced respiratory infections in healthy as well as hospitalized children [34].

Asthma is a major potential target for probiotics, due to its frequency, related pathogenesis, and the lack of consistently effective preventive strategies. However, data on early intestine microbial manipulation are missing, because asthma develops progressively until school age, and the long-term follow-up of probiotic and prebiotic study is limited.

Manipulation of gut microbiota in CF by changing dietary content of indigestible carbohydrate and short-chain fatty acids, namely, butyrate, may improve undernutrition and play an anti-inflammatory effect on animals [35]. Restoration of gut microbiota by probiotics improves nutritional status, energy intake, and respiratory function in cystic fibrosis [35]. Three RCT concluded that probiotic administration in particular LGG and *Lactobacillus reuteri* has been related with a reduction of episodes of pulmonary exacerbations in children with CF [36–38]. The first evidence of the potential benefits of probiotic administration in CF came from a prospective randomized placebo-controlled crossover trial performed in two groups of patients with CF chronically colonized by *Pseudomonas aeruginosa*. Nineteen children were given LGG for 6 months followed by placebo (oral rehydration solution) for the subsequent 6 months. In parallel, 19 children were given the placebo during 6 months then the probiotic for the same period of time. The patients on LGG had a significant reduction of intestinal inflammation and of episodes of pulmonary exacerbations and hospitalization rates, with a decrease in IgG, suggesting that there is a relationship between intestinal and pulmonary inflammation. The intake of this probiotic was associated with a significant increase of the maximal forced expiratory volume in 1 second (FEV1) compared to the placebo as well as to a significant increase of body weight [36]. In another prospective randomized, double-blind, placebo-controlled study, 61 patients with CF were randomly assigned to receive 10^10^ colony-forming units *Lactobacillus reuteri* per day or placebo for 6 months. Pulmonary exacerbations and the number of upper respiratory tract infections were significantly reduced in the treatment group compared with the placebo group [39].

As for bronchopulmonary dysplasia, no studies on humans have been conducted with pre- or probiotics. Moreover, in intermittent hypoxia induced in animal models, the supplementation with probiotics may improve pulmonary status, by acting on the specific matrix metalloproteinases suggesting a beneficial effect on lung inflammation. In addition, prebiotic uses have been associated with arrested lung vascular endothelial growth factor signaling highly involved in lung microvascular development, suggesting preservation of angiogenesis [40].

However, the effects of probiotics are likely to be time-dependent, indicating the need for comparative clinical trials evaluating early-life microbiota modulation impact on lung health status. In particular, future studies are required in order to analyze how gut microbial composition in early life influence lung disease occurrence and history, investigating also the role of functional foods in preventing and/or modifying lung health status in children.

## Figures and Tables

**Table 1:** Main factors and phases of early microbial imprinting.

(1) Prenatal (colonization with vertically transferred microbial material)
(2) Delivery (cesarean section versus natural)
(3) Feeding (human milk versus formula milk)
(4) Infections
(5) Antibiotics
(6) Intestinal environment (cystic fibrosis, short gut, celiac disease)

**Table 2:** Gut microbiota alterations induced by antibiotic use.

Antibiotics	Gut microbiota alterations	Study	References
Ciprofloxacin	Decrease of the taxonomic richness, diversity, and evenness of the community	Healthy adult humans	[41, 42]
Amoxicillin Cefoperazone	Long-lasting alterations in the gut microbial community including a decrease in overall diversity	Young mice	[43]
Ampicillin Vancomycin Metronidazole Neomycin	Decrease of microbial diversity and colonization with antibiotic-resistant microbes	Young mice	[44]
Ceftriaxone	Gut microbiota dysbiosis	Adult mice	[45]
